# Bayesian inference of a spatially dependent semi-Markovian model with application to Madagascar Covid’19 data

**DOI:** 10.1371/journal.pone.0326264

**Published:** 2025-07-07

**Authors:** Angelo Raherinirina, Stefana Tabera Tsilefa, Tsidikaina Nirilanto, Solym M. Manou-Abi

**Affiliations:** 1 Centre de Recherche sur l’Enseignement des Mathématiques, Ecole Normale Supérieure, Fianarantsoa, Madagascar; 2 Laboratoire Informatique et Mathématiques Appliquées pour le Développement, University of Fianarantsoa, Fianarantsoa, Madagascar; 3 Institut Montpelliérain Alexander Grothendieck, Montpellier, UMR CNRS 5149, France; 4 Laboratoire de Mathématiques et Applications, Université de Poitiers, UMR CNRS 7348, France; Federal University of Pernambuco: Universidade Federal de Pernambuco, BRAZIL

## Abstract

This article presents an approach to stochastic analysis of disease dynamics. We develop an explicit semi-Markovian model that accounts for spatial dependence, operating in discrete time over a finite state space. The model allowed us to have a propagation model conditioned by neighboring states and quantifies two key characteristics : spatial propagation timescales and propagation law in a region dependent on neighboring states. The model is inferred from data collected on the spread of Covid’19 in Madagascar’s 22 regions, using the Bayesian approach to get a better idea of model parameter values. The result has demonstrated the effect of neighborhoods on the propagation dynamics of diseases. We conclude with a discussion of potential future theoretical developments.

## 1 Introduction

Following the emergence of Covid’19 and its spread around the world, many countries have implemented multiple interventions, including non-pharmaceutical and pharmaceutical interventions. The Malagasy government has launched awareness, protection and intervention programmes and campaigns. The observed epidemic data studied in this paper relate to the analysis of the spread of the Covid’19 epidemic during the first wave of the epidemic, which began in March 2020 and ended in October 2020, and was reported in all the *R* = 22 regions of the island of Madagascar. Many mathematical models of the Covid’19 coronavirus epidemic have been developed over many years [1,2]. To model phenomena that evolve over time, such as the evolution of a small epidemic, it is often appropriate to represent it by a stochastic process. The Markov model is widely used in epidemiology when working in a discrete state space [3–5] and also in environmental and control analysis for land use dynamics [6–11]. The power and efficiency of the Markovian approach in analysing and modelling complex systems has been known for decades [12,13]. In its various forms and extensions, the use of Markov models has become intuitive and natural for dealing with sequence data, including in epidemiology [1,14], the work of Linda J.S. Allen being one of the best references on this point [15,16]. The most natural model is that of a classical Markov chain, which imposes that the sojourn times in each state are geometrically or exponential distributed [10,11,17]. The adequacy tests performed in this study indicate that this assumption does not always hold for the observed data. A semi-Markov model may be appropriate when the sojourn times laws in different states are not geometric or exponential [7,18,19]. The results of preliminary tests on sojourn times allow us to consider other alternatives such as discrete Weibull and Poisson distributions. In addition to suitability tests on the sojourn times, we also perform spatial dependence tests to check the spatial proximity of the observed state transitions [20]. In this study, we will operate on this aspect by integrating a spatial parameter for the state transition according to the neighbouring observations. These models were introduced by [21] in order to propose new strategies for analysing the evolution of the spatial (joint) distributions of revenues in time and space, using this spatialised Markovian extension to measure the spatial dependence of a given dynamic. The semi-Markov hypothesis makes the models more flexible and tractable [22,23]. In this paper, we try to model the dynamics of Covid’19 from its arrival in Antananarivo (the capital) in March 2020 to its appearance in the other regions of Madagascar. By plotting the intensity rates of the appearance of Covid’19 in each region, we can study the dynamics of its spread in a given region according to the neighbouring regions. In this approach, we propose a classification of the intensity rates into three levels: low, medium and high. Based on the adequacy tests conducted for these three states, the classical Markov assumption appears not to always hold. This motivates the use of semi-Markov models, which provide a natural extension of classical Markov models by allowing greater flexibility in the modeling of sojourn times. We will also address a spatial extension of the semi-Markov model. The remainder of the paper is organized as follows. First, the data-driven model selection process by performing statistical tests on the fit of the data to the sojourn time laws and the spatial dependence of the observations. The results of the statistical tests then lead us to the spatialisation of the semi-Markovian model. Secondly, we perform statistical inference on the model parameters. To do this, we use a Bayesian approach to overcome the data size constraint using expert knowledge. Finally, we discuss the estimation and simulation results of the model and highlight future theoretical and future work.

## 2 Material and methods

**Fig 1:**
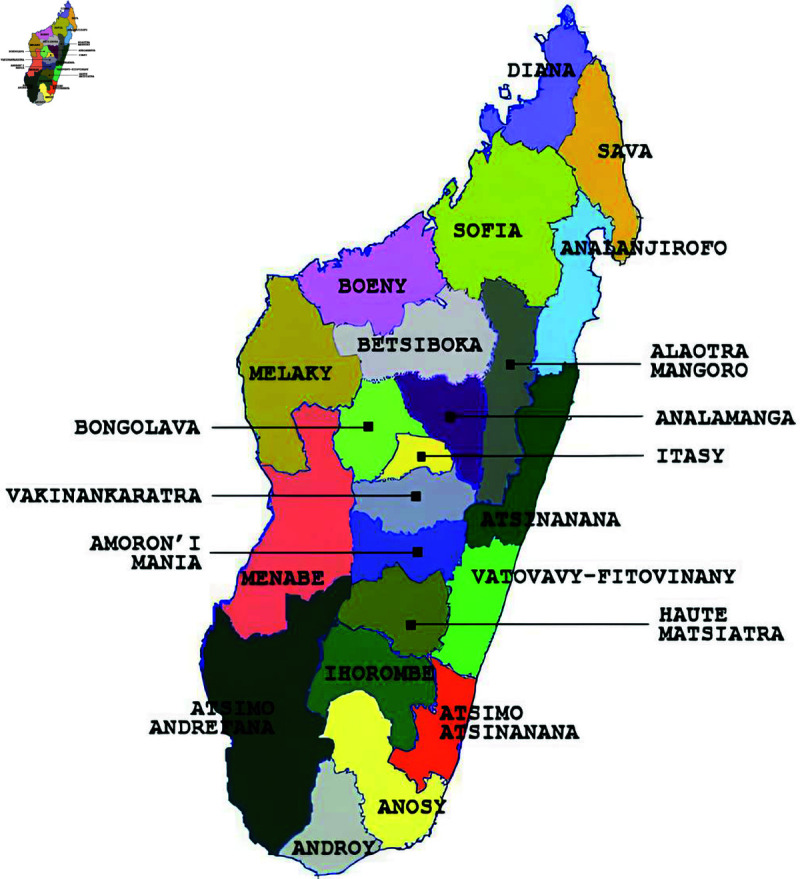
Map of the 22 regions of Madagascar.

The methods outlined below are based on the daily number of Covid’19 cases during the first wave of the pandemic, spanning March to October 2020, across the 22 regions of Madagascar presented in Fig 1.

### 2.1 Data

**Fig 2:**
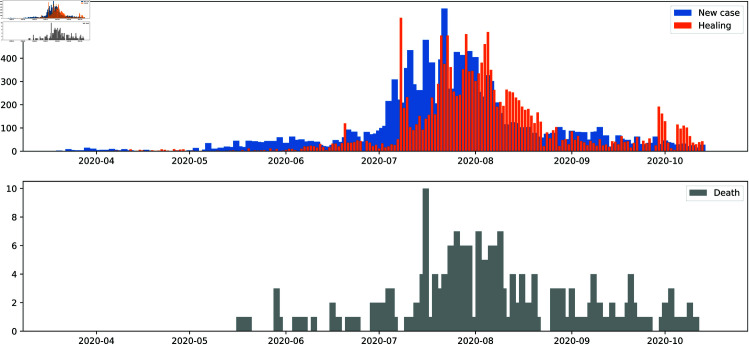
Daily number of cases during the first wave in Madagascar (“ https://ourworldindata.org/coronavirus-data”).

The daily number of new cases reported during the above study period is shown in Fig 2.

**Fig 3:**
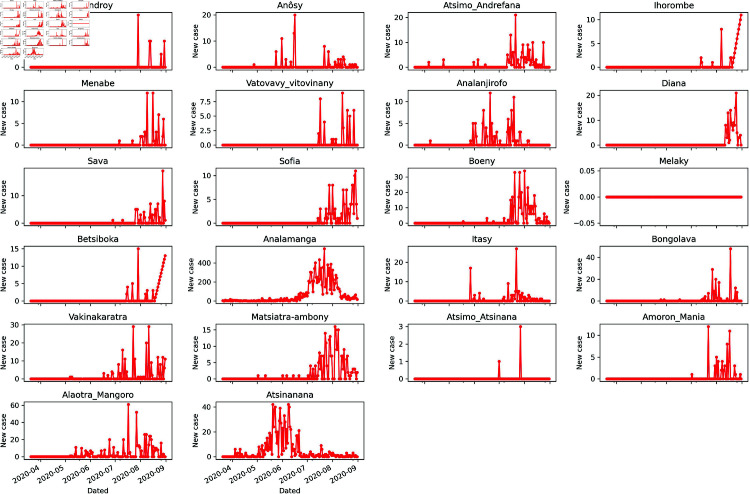
Daily confirmed cases in Madagascar’s 22 regions.

The spatio-temporal evolution of confirmed cases across these regions is presented in Fig 3. To compute the frequency distribution of the cases for each region, we will also consider the population size as well as the total number of cases cumulated.

### 2.2 Markov models and spatial dependency

Markov models are the most popular form of analytical framework for statistical models used to describe systems transition from one state to another in a state space(finite). These models are widely used in various fields, such as epidemiology, engineering, decision-making processes and so on. They are simple, well-suited to systems with clear state transitions and computationally efficient, with sound theoretical foundations and well-established analysis methodology. Preliminary results presented in [20] used transition matrices with spatial dependency and neighborhood-conditioned sojourn time distribution to test whether there is a significant difference in spatial lag, determined by the states of neighboring regions. This earlier study highlighted the importance of incorporating spatial dependence to accurately model propagation dynamics, with the difference of sojourn time laws and independence of neighborhood in sojourn times concluding the relevance of a non-Markovian model. On this basis, the present paper adopts semi-Markovian models with spatial dependency in state transition. Maximum likelihood estimation (MLE) is a point estimator used to estimate the appropriate parameters given the observed data. In contrast, the Bayesian method [24] incorporates prior information about the parameters by estimating their distribution. It is widely recognized that the Bayesian approach often provides more accurate results than MLE. The following outlines some key methodological considerations.

For a given region r∈{1,…22}, let *n*_*rj*_ denote the number of Covid’19 cases recorded during the *j*-th observation week (j∈{1,…,21}, with 21 weeks of observation in our dataset). The intensity of Covid’19, denoted by ${\text{Ic}}_{rj}$, for region *r* during week *j* is defined as (see [20,25]) :


$${\text{Ic}}_{rj}=\frac{n_{rj}}{\sum_{j=1}^{21}n_{rj}/21}.$$

We then highlight three distinct state profile observations as follows. The choice of the threshold, as described in Table 1, corresponds to the statistical position parameter, ensuring an approximately homogeneous distribution of states. This approach allows for the classification of regions into comparable categories based on their epidemic intensity.

**Table 1:** The proposed intensity classification of Covid-19.

Covid-intensity (Ic) range	Covid-intensity level	Covid-intensity code or state
Ic≤0.5	Low	L (Class 1)
0.5<Ic≤1.5	Medium	M (Class 2)
Ic>1.5	High	H (Class 3)

Consider a stochastic process $(Z_{n}{)}_{n\in \mathbb{N}}$ with value in this state space, denoted by E. For a state i∈E, *T*_*i*_ defines the sojourn time in state *i* and is given by $T_{i}=\inf\{n\in \mathbb{N};Z_{n}\neq i|Z_{0}=i\}$. After identifying the different types of sojourn time distributions, as detailed in the next section, we assume that the distribution of sojourn times in the LowLow state depends on the nature of the transition originating from that state. More precisely, we assume that for transitions from the LowLow state to the HighHigh state, the sojourn times follow a discrete Weibull distribution, whereas for transitions from the LowLow state to the MediumMedium state, the sojourn times follow a Poisson distribution.

#### 2.2.1 Spatial dependency analysis.

**Fig 4:**
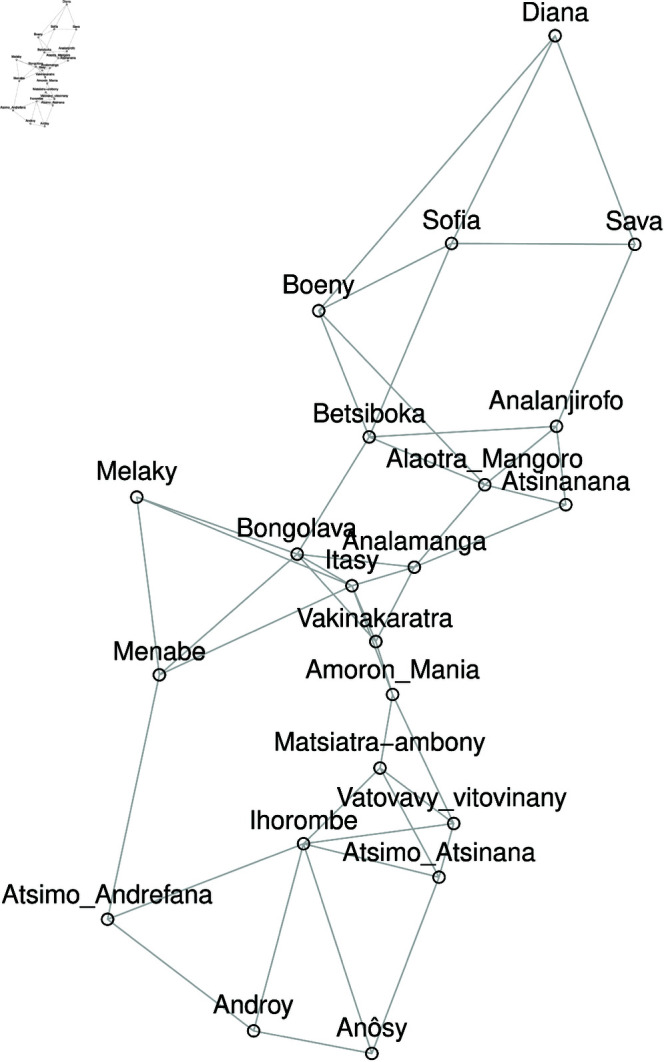
Neighborhood identification by nearest neighbor method (closest neighbor of order k=3).

We are concerned here with spatial homogeneity tests, which consist in identifying some geographical areas of Madagascar according to the intensities of their spatial neighbors. The given *k* nearest neighbors of the structure in terms of intensity [20,26] is given in Fig 4 for *k* = 3. The Markov transition matrix can be modified so that the transition probabilities of a given region depend on the intensity class of its spatial neighborhood. Rey [21] developed a theoretical framework that expanded the conventional Markov chain to include the influence of neighbors (spatial dependence) on the progression of the distribution and the probability of transition, incorporating a spatial delay. The first column of Table 2 displays the state of the spatial lag (space neighbor), i.e. the state in which the average Covid’19 intensity per capita of the neighbors of a region belongs to it. The transition probability from the neighboring class *i* to the neighbouring class *j*, given that the neighbors are in class *v*, is denoted by $p_{ij}/(v)$. This results in a three-dimensional probability hypermatrix ${\hat{P}}^{\left(v\right)}$ corresponding to the transition matrices of the process conditioned by the neighborhood states [21].

**Table 2:** The spatial Markov matrix of Rey [21].

Space neighbour	$t_{i}$	$t_{i+1}$		
		class 1	class 2	class 3
Class 1	Class 1	*p*_11/1_	*p*_12/1_	*p*_13/1_
	Class 2	*p*_21/1_	*p*_22/1_	*p*_23/1_
	Class 3	*p*_31/1_	*p*_32/1_	*p*_33/1_
Class 2	Class 1	*p*_11/2_	*p*_12/2_	*p*_13/2_
	Class 2	*p*_21/2_	*p*_22/2_	*p*_23/2_
	Class 3	*p*_31/2_	*p*_32/2_	*p*_33/2_
Class 3	Class 1	*p*_11/3_	*p*_12/3_	*p*_13/3_
	Class 2	*p*_21/3_	*p*_22/3_	*p*_23/3_
	Class 3	*p*_31/3_	*p*_32/3_	*p*_33/3_

The spatial homogeneity test is conducted using the previous *k* = 3 nearest neighbours, with spatial autocorrelation indices measuring the spatial dependence between values at different locations in space. The Moran diagram facilitates the interpretation of the spatial structure see [27]. The Local Moran’s I statistic quantifies the degree of spatial autocorrelation around each spatial unit within the study area. It serves as a Local Indicator of Spatial Association (LISA), capturing localized clustering patterns of similar or dissimilar values. Formally, it is defined as:


$$I_{i}=\frac{R}{\sum_{j=1}^{n}(A_{j}-\bar{A}{)}^{2}}(A_{i}-\bar{A})\sum_{j=1}^{n}w_{ij}(A_{j}-\bar{A})$$

where:

- *R* is the total number of spatial units (regions),
- *i* and *j* are indices corresponding to two different spatial units (i,j∈{1,…,R}),
- *A*_*i*_ and *A*_*j*_ denote the observed values at locations *i* and *j*, respectively,
- $\bar{A}$ is the mean of the observed values across all locations,
- We transform the link between points *i* and *j* into the element *w*_*ij*_ of the weight matrix *W*.
  $$w_{ij}=\left\{ \begin{array}{cc} 1 & \text{if i and j are considered neighbours}\\ 0 & \text{otherwise} \end{array}\right.$$
- The weight matrix quantifies the spatial correlation between geographical areas through the neighbourhood matrix denoted *W*.

The numerator measures the spatial cross-product of deviations from the mean, weighted by spatial proximity, while the denominator normalizes this by the overall variance of the observations. The value of the global Moran’s *I* statistic lies between –1 and 1. A value close to 1 indicates strong positive spatial autocorrelation (similar values cluster together). A value close to –1 indicates strong negative spatial autocorrelation (dissimilar values are neighbours) and a value near 0 suggests spatial randomness.

#### 2.2.2 A semi-Markovian model with spatially dependence.

We denote by ${ℳ}_{E}$ the set of real matrices on E×E and by ${ℳ}_{E}(\mathbb{N})$ the set of matrix-valued functions defined on ℕ, with values in ${ℳ}_{E}$. Consider a discrete-time stochastic process $(Z_{n}{)}_{n\in \mathbb{N}}$ on the probability space (Ω,ℱ,ℙ) with value in *E* that describes the state of the system through the following tools:

1. The discrete process $J=(J_{n}{)}_{n\in \mathbb{N}}$ with state space *E* measures the n-th jump time.
2. The discrete process $S=(S_{n}{)}_{n\in \mathbb{N}}$ represents the successive time points at which state transitions occur in $(Z_{n}{)}_{n\in \mathbb{N}}$.
3. The chain $X=(X_{n}{)}_{n\in \mathbb{N}}$ with state space ${\mathbb{N}}^{*}$, $X_{n}:=S_{n}\;-\;S_{n-1}$ describes the sojourn time in state *J*_*n*−1_.

The matrix-valued function **q**=$\left(q_{ij}(k))\in {ℳ}_{E}(\mathbb{N}\right)$ is a discrete-time semi-Markov kernel define by :


$$q_{ij}(k):=\mathbb{P}(J_{n}=j,\;S_{n}-S_{n-1}=k|J_{n-1}=i).$$

It denotes the probability of transitioning from state from a state *i* to another state *j* after a stay of *k* time units in *i*. We refer to [28,29] for more details on the construction of Semi-Markov chain. The discrete time process $(Z_{n}{)}_{n\in \mathbb{N}}$ is the semi-Markovian Chain associated with the Markovian renewal chain $(J_{n},S_{n}{)}_{n\in \mathbb{N}}$ if :


$$\mathbb{P}(J_{n}=i_{n},S_{n}-S_{n-1}=X_{n}|J_{0:n-1}=i_{0:n-1},X_{0:n-1}=X_{0:n-1})\;=\mathbb{P}(J_{n}=i_{n},S_{n}-S_{n-1}=X_{n}|J_{n-1}=i_{n-1}),$$

where *X*_0:*n*−1_ denotes $\left(X_{0},X_{1},\ldots ,X_{n-1}\right)$. When Equation is independent of n, the Markov renewal chain (*J*,*S*) is said to be homogeneous. Generally, semi-Markov chains are not Markov processes, as they do not satisfy the Markov property when the sojourn time distribution deviates from a geometric distribution. Furthermore, a semi-Markov process allows arbitrarily distributed sojourn times within any state while preserving the Markov property for the embedded (discrete-time) Markov chain. For more details on the construction of semi-Markov chains, refer to [28,29]. We denote by $P=(p_{ij/(v)}{)}_{i,j,v\in E}$ the transition matrix of (*J*_*n*_), incorporating the spatial conditionality as defined in [20,30]:


$$P_{ij/(v)}:=\mathbb{P}(J_{n+1}=j|J_{n}=i,V_{n}=v),i,j,v\in E,n\in \mathbb{N}$$

We will consider the following assumption for all (i,j)∈E×E:

1. The conditional distribution *f*_*ij*_(.) of $X_{n+1}=S_{n+1}-S_{n},n\in \mathbb{N}$ is independent of the neighborhood state:
  $$f_{ij}(k):=\mathbb{P}(X_{n+1}=k|J_{n}=i,J_{n+1}=j),k\in \mathbb{N}$$
2. The conditional cumulative distribution *F*_*ij*_(.) of $X_{n+1}=S_{n+1}-S_{n},n\in \mathbb{N}$ is given by
  $$F_{ij}(k):=\mathbb{P}(X_{n+1}\leq k|J_{n}=i,J_{n+1}=j)=\sum_{l=0}^{k}f_{ij}(l),k\in \mathbb{N}.$$
  Note that, for all i,j,v∈E and for all k∈ℕ, the following relation holds:
  $$f_{ij}(k)=\left\{ \begin{array}{cc} \frac{q_{ijv}(k)}{P_{ij(v)}},n,k\in \mathbb{N} & \text{si }P_{ijv}\neq 0\\ 1_{\left\{k=\infty \right\}} & \text{si }P_{ij(v)}=0 \end{array}\right.$$ (1)
3. The sojourn distribution time, *h*_*i*_(.), in state *i* is given by :
  $$h_{i}(k):=\mathbb{P}(T_{n+1}-T_{n}=k|Y_{n}=i)=\sum_{j\in E}q_{ij}(k),k\in \mathbb{N}.$$
4. The sojourn cumulative distribution time, *H*_*i*_(.), in state *i* is given by the following formula:
  $$H_{i}(k):=\mathbb{P}(T_{n+1}-T_{n}\leq k|Y_{n}=i)=\sum_{l=1}^{k}h_{i}(l),k\in \mathbb{N}.$$

Note that, from Eq (1), the semi-Markov kernel satisfies the following relation:


$$q_{ij}(k)=P_{ij/(v)}f_{ij}(k)$$

for all i,j∈E and k∈ℕ such that $p_{ij}\neq 0$. It is clear that the changing state is controlled by the following hypermatrix, according to the dependence of the neighborhood region.

$$P_{ij/(v)}=(\begin{array}{ccc} 0 & \theta_{1}^{\left(v\right)} & 1-\theta_{1}^{\left(v\right)}\\ \theta_{2}^{\left(v\right)} & 0 & 1-\theta_{2}^{\left(v\right)}\\ \theta_{3}^{\left(v\right)} & 1-\theta_{3}^{\left(v\right)} & 0 \end{array}),$$ (2)

for i,j∈{L,M,H} and v∈{1,2,3}. and then, the sojourn time distribution is as follows :

$$f(k)=(\begin{array}{ccc} 0 & 𝒫(\lambda ,k) & 𝒲(\eta ,\beta ,k)\\ 𝒢(\gamma_{1},k) & 0 & 𝒢(\gamma_{2},k)\\ 𝒢(\gamma_{3},k) & 𝒢(\gamma_{4},k) & 0 \end{array}),k\in \mathbb{N}$$ (3)

where λ is the parameter of the Poisson distribution, (η,β) represents the parameters of the discrete Weibull distribution, and $\gamma =(\gamma_{1},\gamma_{2},\gamma_{3},\gamma_{4})$ denotes the parameters of the geometric distributions considered.

### 2.3 Bayesian statistical inference for parameter estimation

We consider a Bayesian approach to estimate the parameters described above. The posterior distributions will be computed using the Monte Carlo Markov Chain (MCMC) approximation method, employing algorithms that have proven effective in similar contexts [18]. Specifically, we aim to estimate the elements characterizing the semi-Markov process with spatial effects, including the transition hypermatrix governing state transitions and the conditional sojourn time distributions. To this end, we employ statistical estimation within a Bayesian framework [31,32]. As previously mentioned, the transition dynamics of a semi-Markov process are fully characterized by the transition matrix of the embedded chain *P* and the conditional sojourn time distribution *f* [11]. Let *n*_*r*_ denote the length of the observations associated with region *r* and $e_{k}^{\left(r\right)}$ the observations of the Covid-19 intensity in region *r*. The neighborhood effect can be represented using a hypermatrix as described above. After recording the sojourn times $\left(X_{k}^{\left(r\right)}\right)$ for a given region *r*, we can compute the times of state changes and the censored sojourn time for the last state visited, denoted by $u_{n_{r}}\;=\;n_{r}\;-\;T_{n_{r}}$. Now, denote by ${\text{k}}^{\left(\text{r}\right)}$ the number of state changes observed for region *r*. We introduce the following notations:

- The value *n*_*ij*_(*k*) or
  $$n_{ij}\overset{def}{=}\sum_{l=0}^{k}{\text{1}}_{\left\{J_{l}=i,J_{l+1}=j\right\}}$$
- is defined as the transition number from *i* to *j* (assuming that there is a total of *k* transitions), where *J*_*l*_ is the included chain that govern the succession states of *X*.
- The value
  $$n_{ij/(v)}^{\left(r\right)}\overset{def}{=}\sum_{l=0}^{k^{\left(r\right)}}{\text{1}}_{\left\{J_{l}^{\left(r\right)}=i,J_{l+1}^{\left(r\right)}=j,V_{l}^{\left(r\right)}=v\right\}}$$
- is defined as the transition number from *i* to *j*, knowing that the region in the neighbourhood is *v*, where $J_{l}^{\left(r\right)}$ is the included chain that govern the succession states of *X* and $V_{l}^{\left(r\right)}$ the states of the process in the neighboring regions of *r*.
- The quantity
  $$n_{i/v}^{\left(r\right)}\overset{def}{=}\sum_{j}n_{ij/v}^{\left(r\right)}$$
- is the number of transitions from *i* to any other state, knowing that the neighbouring region is *v* and set $n_{i/v}\overset{def}{=}\sum_{r=1}^{R}n_{i/v}^{\left(r\right)}$.

The empirical estimator of the transition matrix $P_{ij}/(v)$ and the conditional sojourn time distribution *f*_*ij*_ are defined as follows:

$${\hat{P}}_{ij}/(v)={\hat{\theta }}^{\left(v\right)}=\frac{n_{ij}/(v)}{n_{i}/(v)};\;{\hat{f}}_{ij}(k)=\frac{n_{ij}(k)}{n_{i}}\;n_{i}=\sum_{j}n_{ij}.$$

We have


$$\mathbb{P}(J_{0:k^{\left(r\right)}}^{\left(r\right)}=e_{0:k^{\left(r\right)}}^{\left(r\right)},X_{1:k^{\left(r\right)}}^{\left(r\right)}=x_{1:k^{\left(r\right)}}^{\left(r\right)},V_{0:k^{\left(r\right)}}^{\left(r\right)}=v_{0:k^{\left(r\right)}}^{\left(r\right)})=\mathbb{P}(X_{k^{\left(r\right)}+1}^{\left(r\right)}\geq u_{n_{r}}|J_{k^{\left(r\right)}}^{\left(r\right)}=e_{k^{\left(r\right)}}^{\left(r\right)})$$

$$=\prod_{r=1}^{R}\mathbb{P}(J_{1:k^{\left(r\right)}}^{\left(r\right)}=e_{1:k^{\left(r\right)}}^{\left(r\right)},X_{1:k^{\left(r\right)}}^{\left(r\right)}=x_{1:k^{\left(r\right)}}^{\left(r\right)},V_{0:k^{\left(r\right)}}^{\left(r\right)}=v_{0:k^{\left(r\right)}}^{\left(r\right)})$$

$$=\prod_{r=1}^{R}\prod_{l=1}^{k^{\left(r\right)}}\delta_{\text{L}}(e_{0}^{\left(r\right)})\;P_{e_{l-1}^{\left(r\right)},e_{l}^{\left(r\right)},v_{l}^{\left(r\right)}}/(r)\;{\bar{H}}_{e_{k^{\left(r\right)}}^{\left(r\right)}}(u_{n_{r}})$$

where $\delta_{L}$ is the initial law of $(J_{l}{)}_{l\in \mathbb{N}}$ and


$${\bar{H}}_{i}^{\left(r\right)}(k)=\mathbb{P}(X_{k^{\left(r\right)}+1}^{\left(r\right)}>k|J_{k^{\left(r\right)}}=i),\;i\in E,k\in \mathbb{N},$$

corresponds to the survival function associated with the time spent in the last visited state [11]. We assume in the following lines that such survival quantities will be neglected (censored data) [33]. The likelihood function associated with the model is given by

$$L(P,f,r)=\prod_{k=1}^{k^{\left(r\right)}}\prod_{v\in E}\prod_{i,j\in E}[P_{ij/v}{]}^{n_{ij/v}^{\left(r\right)}}\;[f_{ij}(k){]}^{n_{ij/v}^{\left(r\right)}(k)}.$$ (4)

Using the expressions for *P* and *f* in the equations (2) and (3), the likelihood can be written as:

$$L(\theta^{\left(v\right)},\lambda ,\eta ,\beta ,\gamma )=\;\prod_{k=1}^{k^{\left(r\right)}}\prod_{v\in E}(\theta_{1}^{\left(v\right)}{)}^{n_{LM/v}}(1-\theta_{1}^{\left(v\right)}{)}^{n_{LH/v}}(\theta_{2}^{\left(v\right)}{)}^{n_{ML/v}}(1-\theta_{2}^{\left(v\right)}{)}^{n_{MH/v}}(\theta_{3}^{\left(v\right)}{)}^{n_{HL/v}}(1-\theta_{3}^{\left(v\right)}{)}^{n_{HM/v}}\;\left[({𝒫}_{\lambda }(k){)}^{n_{\text{LM}}(k)}({𝒲}_{\left(\eta ,\beta \right)}(k){)}^{n_{\text{LH}}(k)}({𝒢}_{\gamma_{1}}(k){)}^{n_{\text{ML}}(k)}({𝒢}_{\gamma_{2}}(k){)}^{n_{\text{MH}}(k)}({𝒢}_{\gamma_{3}}(k){)}^{n_{\text{HL}}(k)}({𝒢}_{\gamma_{4}}(k){)}^{n_{\text{HM}}(k)}\right]$$ (5)

where $\theta^{\left(v\right)}=(\theta_{1}^{\left(v\right)},\theta_{2}^{\left(v\right)},\theta_{3}^{\left(v\right)})$ is the parameter value of the transition hypermatrix.

#### 2.3.1 Bayesian approach with informative prior distributions for estimating sojourn time laws.

Now, let us proceed to estimate the parameters described above using a Bayesian approach. We assume that the conditional sojourn time distributions are spatially homogeneous but characterized by different parameters, as follows:


$$f_{ij}(k)=(\begin{array}{ccc} 0 & 𝒫(\lambda ,k) & 𝒲(\eta ,\beta ,k)\\ 𝒢(\gamma_{1},k) & 0 & 𝒢(\gamma_{2},k)\\ 𝒢(\gamma_{3},k) & 𝒢(\gamma_{4},k) & 0 \end{array}),k\in \mathbb{N}$$

where λ represents the parameter of the Poisson distribution, (η,β) denotes the parameters of the discrete Weibull distribution, and $\gamma =(\gamma_{1},\gamma_{2},\gamma_{3},\gamma_{4})$ corresponds to the parameters of the geometric distributions under consideration. In the following, we choose to estimate only the parameters λ and γ using a Bayesian approach. This decision stems from the fact that estimating the parameters of the discrete Weibull distribution within this framework would incur significant computational costs. For the geometric distribution parameter, we will use a Beta distribution as an informative prior, since it is defined over the interval [0,1]. For the Poisson distribution parameter, we will adopt a Gaussian distribution as the prior. We can then decompose (4) as follows:


$$L(\theta^{\left(v\right)},\lambda ,\eta ,\beta ,\gamma )=L_{1}(\theta^{\left(v\right)})\;L_{2}(\lambda )\;L_{3}(\eta ,\beta )\;L_{4}(\gamma ),$$

where


$$L_{1}(\theta^{\left(v\right)})=\prod_{v\in E}\;(\theta_{1}^{\left(v\right)}{)}^{n_{\text{LM/v}}}\;(1-\theta_{1}^{\left(v\right)}{)}^{n_{\text{LH/v}}}\;(\theta_{2}^{\left(v\right)}{)}^{n_{\text{ML /v}}}\;(1-\theta_{2}^{\left(v\right)}{)}^{n_{\text{MH/v}}}(\theta_{3}^{\left(v\right)}{)}^{n_{\text{HL/v}}}\;(1-\theta_{3}^{\left(v\right)}{)}^{n_{\text{HM/v}}},$$

$$L_{2}(\lambda )=\prod_{k=1}^{N}({𝒫}_{\lambda }(k){)}^{n_{\text{LM}}(k)},\;L_{3}(\eta ,\beta )=\prod_{k=1}^{N}({𝒲}_{\left(\eta ,\beta \right)}(k){)}^{n_{\text{LH}}(k)},$$

$$L_{4}(\gamma )=\prod_{k=1}^{N}({𝒢}_{\gamma_{1}}(k){)}^{n_{\text{ML}}(k)}({𝒢}_{\gamma_{2}}(k){)}^{n_{\text{MH}}(k)}({𝒢}_{\gamma_{3}}(k){)}^{n_{\text{H L}}(k)}({𝒢}_{\gamma_{4}}(k){)}^{n_{\text{HM}}(k)}.$$

Under the classical framework, the Maximum Likelihood estimators of the parameters are as follows [6]:


$$\hat{\eta }=1-\frac{\sum_{k=1}^{M}kn_{ij}(k)}{\sum_{k=1}^{M}n_{ij}(k)}\;\hat{\beta }=\frac{1}{\log(2)}\log\left(\frac{\log(\hat{\eta })-\frac{\sum_{k=1}^{M}n_{ij}(k+1)}{n_{ij}}}{\log(\hat{\eta })}\right).$$

$$\hat{\gamma }=\frac{\sum_{k=1}^{M}n_{ij}(k)}{\sum_{k=1}^{M}kn_{ij}(k)}\;\hat{\lambda }=\frac{\sum_{k=1}^{M}kn_{ij}(k)}{\sum_{k=1}^{M}n_{ij}(k)}.$$

Now, based on the Bayesian estimation framework, the distribution of an entire parameter vector Σ, conditional on the observations, referred to as the posterior distribution $\pi_{posterior}$ is derived using Bayes’ theorem:


$$\pi_{posterior}\propto L(\Sigma )\pi_{prior}(\Sigma )$$

where L(Σ) is the likelihood function, which is simply the distribution of the observations conditional on Σ.

#### 2.3.2 Bayesian approach with non-informative prior distributions for estimating the transition matrix.

This approach leverages Bayesian inference to incorporate uncertainty in disease transmission dynamics, particularly useful when prior knowledge is limited or data is sparse. For the parameters of the transition hypermatrix of the embedded chain, denoted as $\theta^{\left(v\right)},\;v\in \{L,M,H\}$, we adopt the non-informative Jeffreys prior [31]. This choice ensures invariance under reparameterization and avoids introducing subjective bias, which is critical in epidemiological applications where unbiased estimation is paramount [34]. The Jeffreys prior is derived from the Fisher information matrix $𝕀(\theta^{\left(v\right)})$, which quantifies the expected curvature of the log-likelihood function:


$$\pi_{\text{prior}}^{1}(\theta^{\left(v\right)})\propto \sqrt{\det[𝕀(\theta^{\left(v\right)})]},\;\text{where}\;𝕀(\theta^{\left(v\right)})={\left[{𝔼}_{\theta^{\left(v\right)}}\left(-\frac{\partial^{2}l_{1}(\theta^{\left(v\right)})}{\partial \theta_{k}^{\left(v\right)}\partial \theta_{l}^{\left(v\right)}}\right)\right]}_{1\leq k,l\leq 3},$$

and $l_{1}(\theta^{\left(v\right)})=\log L_{1}(\theta^{\left(v\right)})$ is the log-likelihood of the observed transitions. This prior is particularly advantageous for multinomial transition models, as it automatically adapts to the scale of the parameters.

Applying Bayes’ theorem, the posterior distribution combines the likelihood $L_{1}(\theta^{\left(v\right)})$ with the Jeffreys prior:


$$\pi_{\text{posterior}}^{1}(\theta^{\left(v\right)})\propto L_{1}(\theta^{\left(v\right)})\;\pi_{\text{prior}}^{1}(\theta^{\left(v\right)}).$$

The Bayesian estimator ${\hat{\theta }}^{\left(v\right)}$ is the posterior mean, which minimizes the expected quadratic loss:


$${\hat{\theta }}^{\left(v\right)}=\int_{\Theta^{\left(v\right)}}\theta^{\left(v\right)}\;\pi_{\text{posterior}}^{1}(\theta^{\left(v\right)})\;d\theta^{\left(v\right)}$$

$$=\frac{\int_{\Theta^{\left(v\right)}}\theta^{\left(v\right)}\;L_{1}(\theta^{\left(v\right)})\;\pi_{\text{prior}}^{1}(\theta^{\left(v\right)})\;d\theta^{\left(v\right)}}{\int_{\Theta^{\left(v\right)}}L_{1}(\theta^{\left(v\right)})\;\pi_{\text{prior}}^{1}(\theta^{\left(v\right)})\;d\theta^{\left(v\right)}}.$$

## 3 Results and discussion

**Fig 5:**
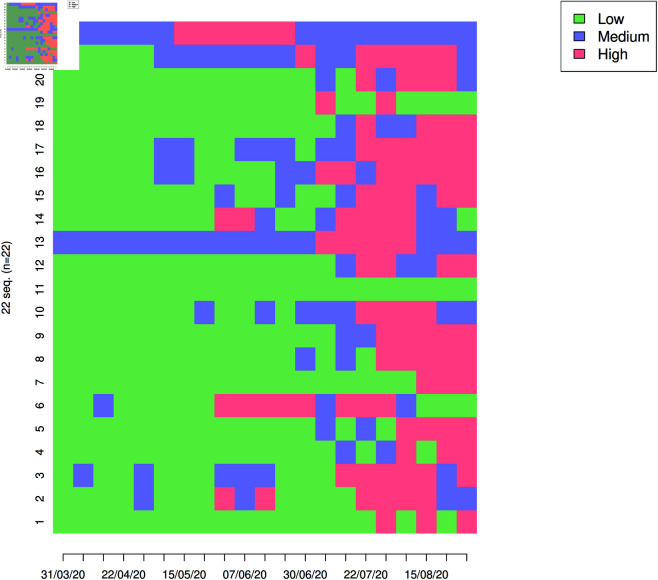
Weekly intensity sequence tables.

**Fig 6:**
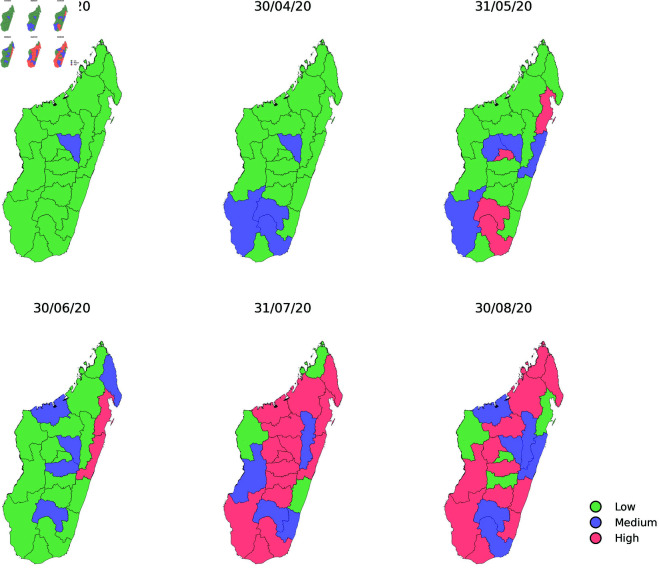
Regional trends recorded for the profiles of the above mentioned three states.

This section begins with a statistical analysis of the Covid’19 epidemic curve on the island of Madagascar using a simple Markov model. The above data seems to reveal that the epidemic exhibited distinct dynamic behaviors across Madagascar’s 22 regions. In our study of COVID-19 intensity, each region is classified into one of three distinct states reflecting increasing levels of epidemic severity: Low (L=1), Medium (M=2), and High (H=3). At any given time *t*, the Covid’19 intensity level variable takes one of these three values, corresponding to a particular state, see Fig 5 for the observed weekly intensity sequence. To provide an overview of the disease’s progression across the country, Fig 6 shows the general trend in the 22 regions during the observation period.

Since the transition between two successive states depends only on the Covid’19 intensity level at the start time, a first-order time-dependent 3-state Markov chain can be constructed. We now turn to justify the choice of our modelling approach based on Markovian testing hypotheses [35].

Note that in the classical Markov assumption it remains to assume that the sojourn-time laws are geometrically distributed [18]. To evaluate such hypotheses, we propose somme Statistical test on the sojourn times laws. For instance, one can use the classical Kolmogorv-Smirnov test [36] to check the adequacy of the geometric law for the sojourn times. The geometric distribution on ${\mathbb{N}}^{*}$ of parameter γ, 0<γ<1 is defined by:


$${𝒢}_{\gamma }(k)=\gamma (1-\gamma {)}^{k-1},k\in {\mathbb{N}}^{*}$$

**Fig 7:**
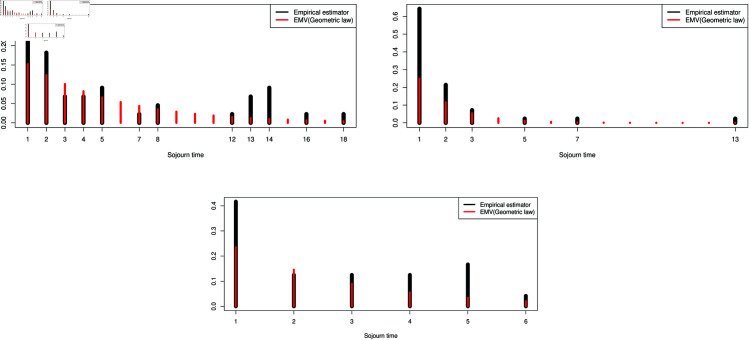
Superposition of the geometric probability distribution (red colour) on the observed sojourn times (black colour).

Fig 7 shows the superposition with a geometric distribution and the results of the goodness-of-fit test in Table 3.

**Table 3:** Goodness-of-fit test.

State	p-value	Goodness-of-fit test
Low	0.1233	hypothesis rejected
Medium	0.001	hypothesis accepted
High	0.007	hypothesis accepted

**Fig 8:**
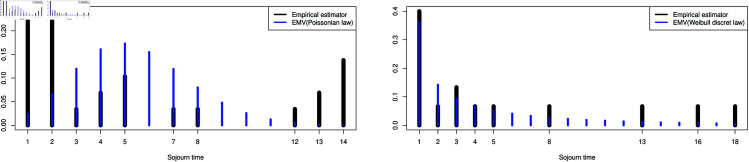
Test on other sejourn times laws.

The above result leads us to examine the following laws for the sejour times laws of the intensity rate. In the case of the state Low, we studied the sejourn time on the states separately according to the type of transition. For the transitions Low→Medium and Low→High, we suggest performing goodness-of-fit tests with other distributions, such as the Poisson distribution and the discrete Weibull distribution as shown in Fig 8. We also present the associated goodness-of-fit tests in Table 4. Recall that the discrete-time Weibull distribution with parameter vector (η,β) is defined as follows:

**Table 4:** Adequacy test result with p-value 0.05.

State	Geometric law	Poisson law	Discrete Weibull law
Low to Medium	hypothesis rejected	hypothesis accepted	hypothesis rejected
Low to High	hypothesis rejected	hypothesis rejected	hypothesis accepted


$$\left\{ \begin{array}{cc} & {𝒲}_{\eta ,\beta }(0)=0\\ & {𝒲}_{\eta ,\beta }(k)=\eta^{(k-1{)}^{\beta }}-\eta^{k^{\beta }}\;\forall k\geq 1 \end{array}\right.$$

where β>0 and η∈[0,1].

**Fig 9:**
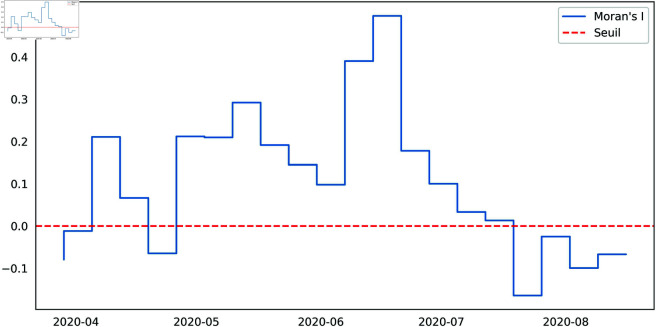
The global indicators of spatial autocorrelation.

**Fig 10:**
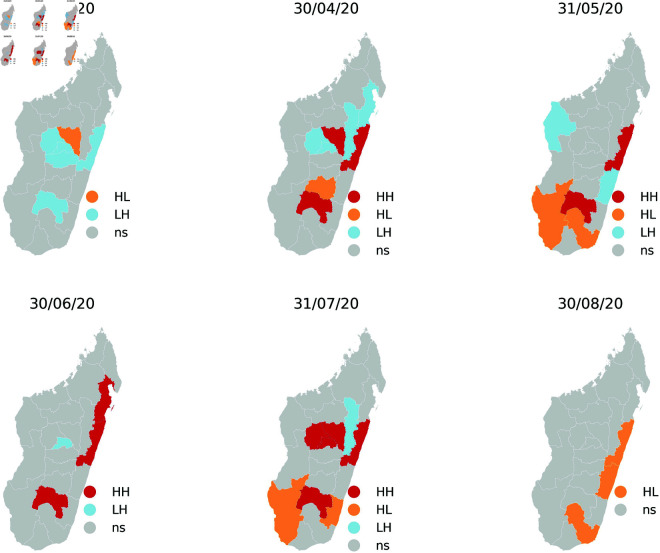
The Local indicators of spatial association map.

In the following lines, we’ll analyse the spatial effect in the transition probability. Building on the previous statement, it is also insightful to consider the use of non-geometric laws for the sojourn times within the states. Therefore, for the rest we will consider a semi-Markovian model in which the transition probabilities of a region depend on its spatial lag. Such spatial dependence will also be analysed. The Moran statistical test, as outlined in Fig 9 for the global spatial autocorrelation and Fig 10 for the Local indicators of spatial association (LISA) has been employed to validate the neighbourhood zones. It demonstrates a p-value of 0.1 when evaluated against the criterion of local spatial autocorrelation.

We can also assess the spatial effect by comparing the elements in Table 2. The influence of neighboring regions becomes evident, for instance, when $p_{23}>p_{23}/(1)$. This indicates that the probability of regions in class 2 (medium intensity) transitioning to class 3 (high intensity) is higher without considering their neighbors is higher than when these regions have low-intensity neighbors. Similarly, if $p_{13}>p_{13}/(1)$, the probability of regions transitioning from class 1 (low intensity) to class 2 (medium intensity) is higher when they have neighbors in class 3 (high intensity) compared to the average transition probability. Conversely, if neighbors exert no influence, the transition probabilities would satisfy the condition $p_{ij}/(1)=p_{ij}/(2)=p_{ij}/(3)$, indicating uniformity of the neighboring regions’ classes [20,37]. The above data analysis show the following. Firstly, the law of the sojourn times on a given state does not ollow a geometric distribution, depending on the nature of the transitions observed. Secondly, the evolution of the intensity of the Covid’19 in a given region depends on the values of the Covid’19 intensity in neighbouring regions.

This means that a semi-Markovian model that includes a spatial effect on the transition matrix could be insightfull. Applying log-likelihood maximization of (5) like that [12,18,38], we obtain :


$${\hat{f}}_{MLE}(k)=(\begin{array}{ccc} 0 & 𝒫(\lambda =5.37,k) & 𝒲(\eta_{1}=0.95,\beta_{1}=5.09,k)\\ 𝒢(\gamma_{1}=0.75,k) & 0 & 𝒢(\gamma_{2}=0.41,k)\\ 𝒢(\gamma_{3}=1,k) & 𝒢(\gamma_{4}=0.31,k) & 0 \end{array}),k\in \mathbb{N}.$$

The Bayesian estimator $\hat{\Sigma }$ of Σ is the mean of the posterior distribution, and it is given by:


$$\hat{\Sigma }=\int \Sigma \pi_{posterior}(\Sigma )d\Sigma .$$

We will use the Markov Chain Monte Carlo (MCMC) method [31,32]. This methods consist in simulating a target probability distribution from an ergodic Markov chain. These techniques were introduced by Metropolis *et al*. [39], they enable numerical sampling according to the a posteriori law. This approach has been previously applied to agrarian dynamics problems [18].

**Algorithm 1. Metropolis-Hastings algorithm with Gaussian kernel: the target distribution is $\pi_{post}\left(\theta \right)$ and the proposal distribution is g(.−θ), where g is the density associated with the centered normal distribution $𝒩(0,\sigma^{2})$.**



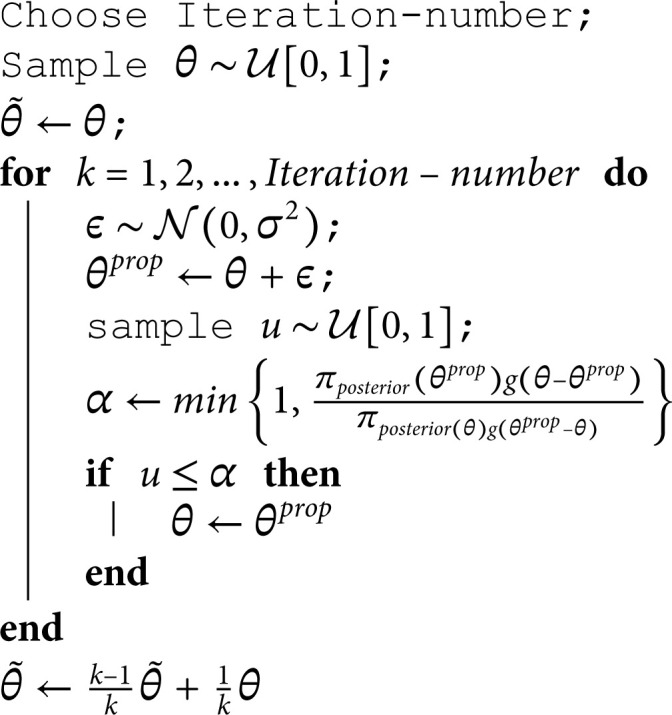



**Fig 11:**
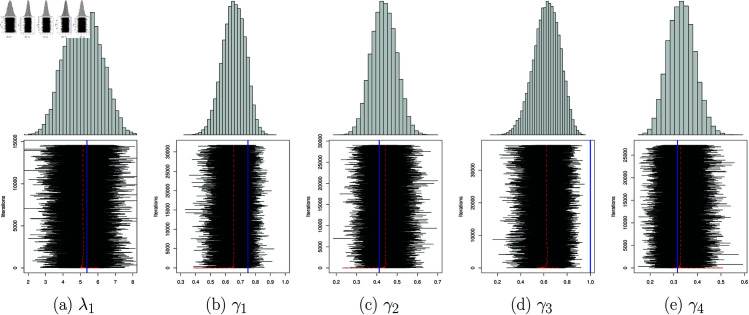
Bayesian estimator of sojourn time parameters: sampling from the posterior distribution using the MCMC method “in black”, the associated mean in “red”, the maximum likelihood estimator in “blue” and the upper graph is the sampling histogram.


Applying the bayesian approach combined with the MCMC method, Fig 11 shows the samples and the evolution of the estimators as a function of iteration. The sojourn time distributions are estimated as follows:

$${\hat{f}}_{Bayesian}(k)=(\begin{array}{ccc} 0 & 𝒫(\lambda =5.02,k) & 𝒲(\eta_{1}=0.95,\beta_{1}=5.09,k)\\ 𝒢(\gamma_{1}=0.654,k) & 0 & 𝒢(\gamma_{2}=0.444,k)\\ 𝒢(\gamma_{3}=0.62,k) & 𝒢(\gamma_{4}=0.33,k) & 0 \end{array}),k\in \mathbb{N}$$

The count of transitions in the dataset is presented in Table 5.

**Table 5:** Number of transition in the data.

Space neighbour	$t_{i}$	$t_{i+1}$		
		class 1	class 2	class 3
Class 1	Class 1	0	8	2
	Class 2	6	0	1
	Class 3	1	1	0
Class 2	Class 1	0	15	3
	Class 2	8	0	7
	Class 3	1	2	0
Class 3	Class 1	0	6	10
	Class 2	7	0	13
	Class 3	4	15	0

**Fig 12:**
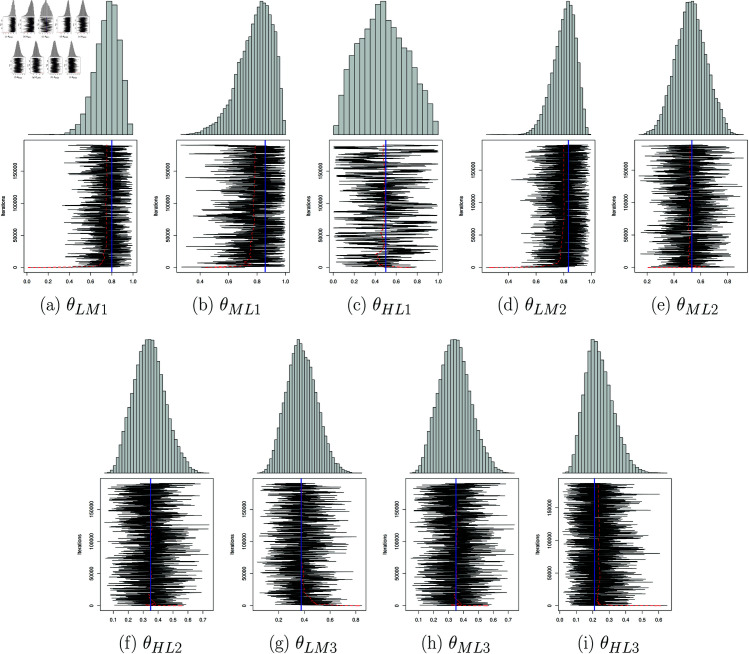
Bayesian estimator and maximum likelihood estimator of the parameter θ of the transition matrix of the spatialized embedded chain: sample of the posterior distribution obtained by MCMC in black, the associated mean in “red”, the maximum likelihood estimator in “blue” and the upper graph is the sampling histogram.

We displayed in Fig 12, the Bayesian estimation ${\hat{\theta }}^{\left(v\right)}$ using Markov Chain Monte Carlo (MCMC) method as well as the Maximum Likelihood estimation.

**Fig 13:**
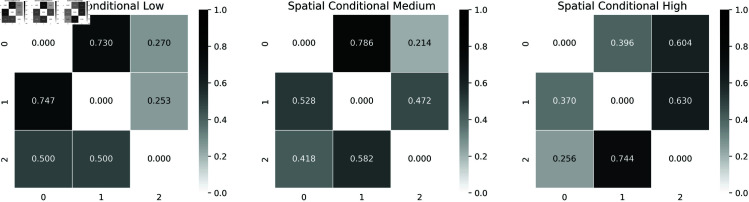
Transition matrix with neighbor dependency.

Bayesian estimators of transition matrixes conditioned on neighborhood states are shown in Fig 13.

### 3.1 Limit laws, simulation and time scale of dynamics

We will first examine the law of limits as conditioned by neighbourhood. With the above estimators, the included Markov chains are all irreducible, and the state sojourn times have finite means. Therefore, the spatial semi-Markov chain admits limit laws conditionally on the neighbors.


$$\Pi_{j}=\frac{\pi_{j}\epsilon_{j}}{\Sigma_{i\in E}\pi_{j}\epsilon_{j}},j\in E$$

where π is the invariant law of the included Markov chain $(Y_{k}{)}_{k\in \mathbb{N}}$ and ϵ the vector containing the mean of the sojour times on the states [33,38,40]. After calculation, we have determined that:


$$\Pi_{|low}=(\begin{array}{ccc} 0.094 & 0.716 & 0.19 \end{array})\;\Pi_{|medium}=(\begin{array}{ccc} 0.057 & 0.649 & 0.293 \end{array})$$

and


$$\Pi_{|high}=(\begin{array}{ccc} 0.126 & 0.498 & 0.376 \end{array})$$

**Fig 14:**
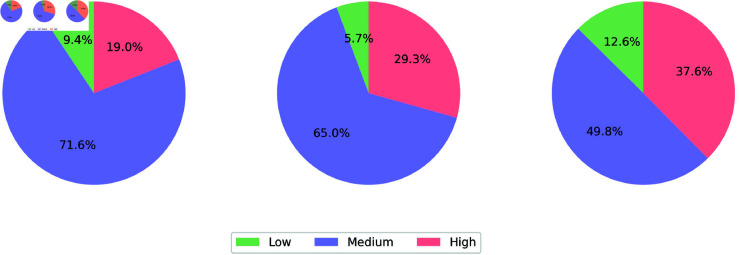
Equilibrium state of the pandemic, conditional on the neighborhood states (low, medium, high).

The limit laws represent the equilibrium state of the pandemic, conditional on the neighboring regions (see Fig 14).

From such results, we observe that once the transition laws stabilize, the Covid’19 cases will predominantly remain in the Medium state, regardless of the state of neighboring regions

#### 3.1.1 Numerical simulation.

Using the numerical simulation method, we can simulate the model over a long trajectory. As the simulated trajectory progresses, the time-scale laws will converge towards a distribution. The simulation method is based on the following algorithm.

**Algorithm 2. Simulation algorithm for the semi-Markov model with spatial dependence.**



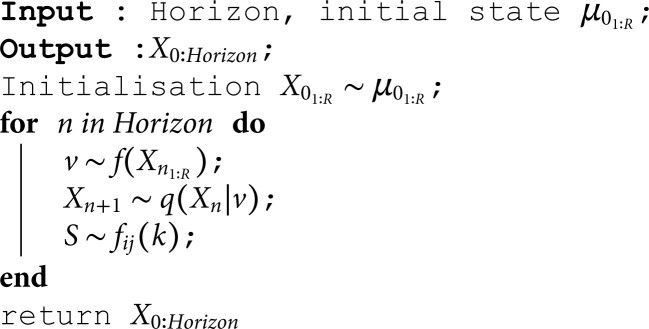



**Fig 15:**
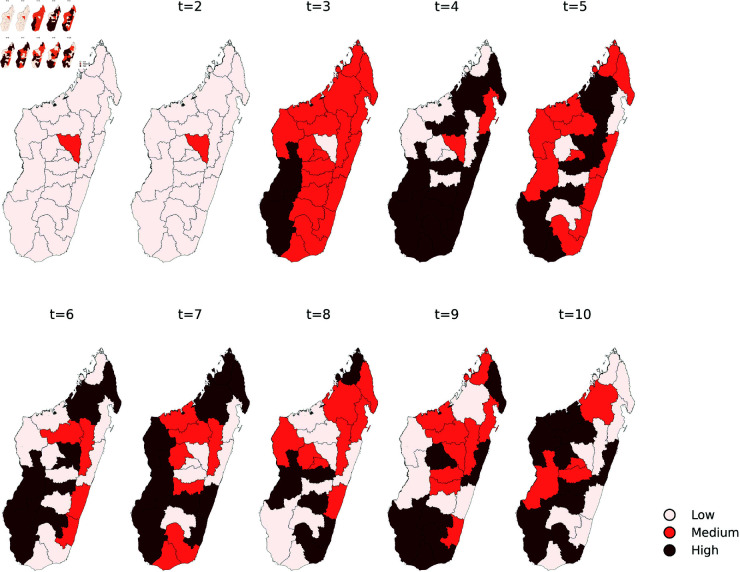
Example for simulation of the proposed model.

This algorithm simulates semi-Markov dynamics with spatial dependency by generating a state trajectory *X*_0:*Horizon*_ over a given horizon for the different regions. Starting from a state i, it identifies the state of influence neighbors v depending on the neighboring states $X_{n_{1:R}}$, then updates the next state j according to a transition law q conditioned by v; and finally it simulates the sojourn time S on i towards j according to a law *f*_*ij*_(*k*). This approach makes it possible to simulate several model trajectories for numerical study after a few evolution time distributions. Fig 15 shows an example of the trajectory of this model.

**Fig 16:**
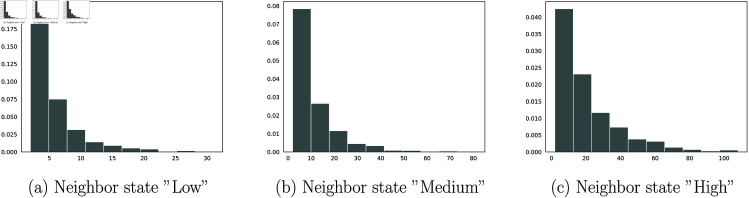
Law on the expected return time to a low state after contamination.

**Fig 17:**
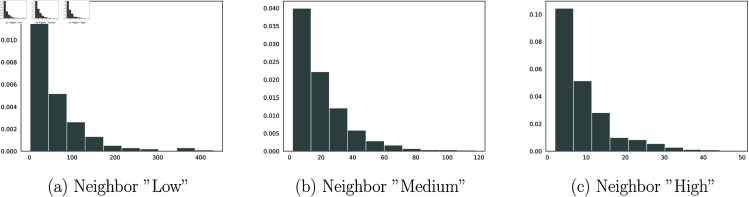
Expected return time to High state after Covid’19 recovery.

Now, we can compute the law of the expected return time for instance to the low state, i.e. the recovery time, and the time to return to the high state, knowing the state of the neighbors based on several simulation samples (see Figs 16 and 17).

For the recovery time following contamination in a region with “low” intensity neighbors, the expected return time is 5.45 weeks. In contrast, for a region with “Medium” intensity neighbors, the expected recovery time is 10.95 weeks, and for a region with “High” intensity neighbors, the the expected recovery time is 19.66 weeks.

For the return to epidemic state after recovery in a region with “low” intensity neighbors, the expected time is 62.95 weeks. For a region with “Medium” intensity neighbors, the expected return time is 19.47 weeks, while for a region neighboring “High” intensity areas, the return time is 9.03 weeks. To conclude this section we conducted a simulation of the spread of the epidemic in space.

**Fig 18:**
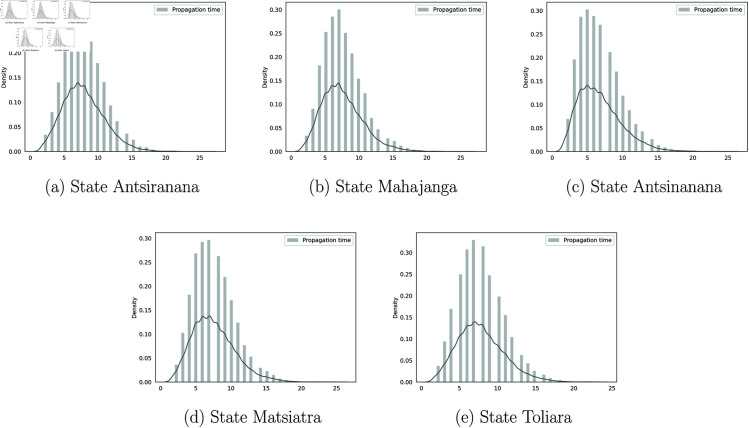
The simulation both in time and space. The line represents the kernel estimate of the empirical distribution.

The Covid’19 began in Antananarivo, the capital city and from the international airport and then spread across Madagascar. Using simulation methods, we estimated the expected duration for each phase of propagation. The results are shown in Fig 18. It was observed that, after the epidemic reached the capital, the province of Atsinanana would transition to the “High” state most rapidly, with an expected duration of 6.7 weeks. In contrast, the duration in the other provinces showed little variation: Antsiranana reached this state in 7.87 weeks, Mahajanga in 7.53 weeks, Matsiatra Ambony in 7.96 weeks, and Toliary in 7.81 weeks.

## 4 Conclusion and perspectives

This study focuses on spatializing a semi-Markovian dynamic, with particular attention to the intensity conditions of neighboring regions. In our model, the spatial component is integrated by considering the states of neighboring areas, with a specific application to the dynamics of Covid’19 in Madagascar. We began by conducting statistical tests to assess our hypotheses. The semi-Markovian nature of the dynamics was confirmed through a goodness-of-fit test for the geometric distribution, which was rejected for certain state residence times. Regarding the influence of neighboring regions, statistical tests based on the global and local Moran’s index confirmed the dependence of several regions on their three nearest neighbors. The parameters of the spatially dependent semi-Markovian model were estimated using both maximum likelihood maximization and a Bayesian approach. These methodologies provided precise estimates, reinforcing the reliability of our model to reflect the epidemiological reality in Madagascar. By estimating the spatial transition matrix conditioned on neighbors, where the state of a region is determined by the maximum intensity of its three nearest neighbors, and confirming this spatial dependence through a homogeneity test, we constructed a model characterized by the hypermatrix of the embedded chain and the laws of residence times. The model’s analysis led to key insights regarding the spread of Covid’19 in Madagascar, particularly the temporal scales of propagation and spatial diffusion across the country. In conclusion, our study introduces an innovative approach to modeling dynamic systems using a spatially dependent semi-Markovian model. The statistical tests confirmed the validity of our model from both the semi-Markovian and spatial perspectives. The results offer valuable insights into the spread of Covid’19, with both temporal and spatial applications in Madagascar. These findings can guide public health policies and help implement targeted strategies in regions that are highly influenced by their neighbors. Looking ahead, we plan to investigate a deep understanding of a kind of spatial Markov model including a mathematical analysis of their asymptotic behavior. In addition, more realistic distance measures to improve the accuracy of our modeling approach. These future developments could contribute to a deeper understanding of spatial Markov models in decision-making to manage transitional dynamics in various contexts.
